# A Study on N_2_O Measurement Characteristics Using Photoacoustic Spectroscopy (PAS)

**DOI:** 10.3390/s140814399

**Published:** 2014-08-07

**Authors:** Soyoung Kang, Seoungjin Kim, Seongmin Kang, Jeongwoo Lee, Chang-Sang Cho, Jea-Hwan Sa, Eui-Chan Jeon

**Affiliations:** 1 Cooperate Course for Climate Change, Sejong University, Seoul 143-747, Korea; E-Mails: kangsso171@naver.com (So.Kang); kimseungjin8611@gmail.com (Se.Kim); smkang9804@gmail.com (Se.Kang); leejwsky@gmail.com (J.L.); changsang-@hanmail.net (C.-S.C.); 2 Environment & Energy Institute, Sejong University, Seoul 143-747, Korea; E-Mail: goodmrsa@gmail.com; 3 Department of Environment & Energy, Sejong University, Seoul 143-747, Korea

**Keywords:** greenhouse gas, nitrous oxide, photoacoustic spectroscopy, gas chromatography

## Abstract

N_2_O, which is emitted mainly from nitrogen decomposition via bacteria, livestock manure, agricultural fertilizer use, fossil fuel combustion and waste incineration, is classified as a substance that causes significant destruction of the ozone layer. The N_2_O measurement methods for these emission sources may be divided into chromatography, optical, and electrical current measurements. Chromatography has been widely utilized for analyzing N_2_O. However, up until now, few studies have been conducted on N_2_O using photoacoustic spectroscopy. Therefore, this study aimed to evaluate performance of photoacoustic spectroscopy in this regard based on laboratory and field test results. The repeatability of photoacoustic spectroscopy was measured at 1.12%, which is lower than the repeatability of 3.0% suggested by the ISO 1564 standard, so, it has shown an excellent repeatability. The detection limit was determined to be 0.025 ppm, and the response time was confirmed to be 3 min and 26 s. The results of comparison between these measurements and GC show that the latter has superior accuracy, but mobility and convenience are superior for PAS. On the contrary, GC has a continuous measurement limitation, but PAS makes it possible to conduct continuous measurements. Therefore, PAS can be extremely useful to confirm the characteristics of N_2_O emissions and to quantify their amount.

## 1. Introduction

As the severity of environmental problems (including abnormal changes in weather and atmospheric pollution caused by climatic change) continues to increase, countries worldwide are undertaking various endeavors to establish goals for greenhouse gas reduction in order to resolve the problems associated with climatic change. The Korean Government has established and announced a voluntary reduction goal of 30% compared to the anticipated Business As Usual (BAU) greenhouse gas emissions by 2020, and now is implementing a Target Management Scheme (TMS) for greenhouse gas emissions and a Renewable Portfolio Standard (RPS) to attain the goal.

In 2010, the total emission of greenhouse gases in the world was 50.1 Gt CO_2_eq, which is an increase of 4.4% compared with the approximate 48 Gt CO_2_eq recorded for 2009. Among these pollutants it was surveyed that N_2_O accounts for 6.3%, which is smaller than the amount of CO_2_ emission that represents 83% of greenhouse gas emissions. However, N_2_O is classified as a substance that causes significant destruction of the ozone layer [1–3]. Its atmospheric concentration has increased by approximately 20% since The Industrial Revolution [4] and the amount has increased more quickly than that of other greenhouse gases. The Global Warming Potential (GWP) of N_2_O is 310 times higher than that of CO_2_, which suggests that the effect of reduction effect would be larger than for other greenhouse gases, so it is judged to require systematic measurement and control [5–7].

N_2_O is mainly emitted by nitrogen decomposition caused by bacteria, livestock manure, agricultural fertilizer use, fossil fuels, and waste combustion [8]. N_2_O levels from these emission sources are measured using Photoacoustic Spectroscopy (PAS), Quartz-enhanced Photoacoustic Spectroscopy (QEPAS), Non-Dispersive Infrared (NDIR), Gas Chromatography (GC), *etc*. PAS, QEPAS and NDIR are mainly utilized to continuously monitor emission concentrations from emitting sources, and GC is used as a discontinuous method that allows the analysis of exhaust gas samples collected from the sources. Many studies on N_2_O utilizing GC have been conducted in Korea [9,10], but no serious evaluation of PAS for the analysis of the N_2_O has been conducted to date. Therefore, this study aimed to find out the characteristics of the analysis results according to the difference between analysis methods by using the PAS and GC methods together.

## 2. Study Methods

### 2.1. N_2_O Measurement Methods and Equipment Conditions

#### 2.1.1. Photoacoustic Spectroscopy (PAS Method)

PAS utilizes the photoacoustic effect principle, which was first discovered by Alexander Graham Bell in 1880 [11]. The principle is the phenomenon that when a modulated light is projected with a constant cycle onto an absorbing medium, an acoustic signal with the same cycle is produced in the gas layer adhered to the material [12]. Since photoacoustic spectroscopy was first applied to gases by Viengerov (1938) and Luft (1943), the method has been widely utilized for gas analysis and vapor detection [13,14].

The PAS method makes it possible to measure almost all substances, including not only gases, liquids, and solid samples, but also specimens in bulk, powder, and gel states. The method is also utilized in the detection of substances with an infinitesimal level (ppb) in the air [15]. The instrumentation is easy to move and portable, providing cost savings, and the possibility of performing measurements in real-time [16]. However, it is very susceptible to vibrations and noises from the surroundings making it very difficult to conduct measurements in places with severe vibrations [17].

In this study, a LSE N_2_O-4405 instrument (LSE Monitors, Groningen, The Netherlands) was utilized as the analyzer, and the analysis conditions were as listed in Table 1. The sample inlet flow rate into the analyzer were set at 80 mL/min, the temperatures of the measurement cell and sample were set at 35 °C and 5∼25 °C, respectively, and the scan ranges were set at 1277.847∼1279.548 cm^−1^. It has also been reported that LSE N_2_O-4405 is affected by interfering materials such as H_2_O, CO_2_, SO_2_, *etc*. [7]. Therefore, the influence from interfering materials was minimized by a connecting molecular sieve (Sigma-Aldrich, St. Louis, OK, USA) trap and Chemisorb S (Bete, Gentbrugge, Belgium) trap to the front terminal of the sample-injecting part in order to remove interfering materials. The detailed analysis conditions of PAS are listed in Table 1.

#### 2.1.2. Gas Chromatography (GC Method)

The GC method analyzes qualitatively and quantitatively by injecting the sample into a flow of gas, then separating the component of the specimen in the flow, and detecting the ingredients [18]. The GC method has a high sensitivity, a superior quantitative reproducibility, and superior heat stability. The method works well for the analysis of stable substances, but it is difficult to analyze unstable chemicals such as intermediates and radicals, and is limited for continuous measurements [14,17].

An Electron Capture Detector (ECD) is a typically utilized detector in the measurement of N_2_O. The GC used in this study was a cp-3800 (Varian, Santa Clara, CA, USA) and the analysis conditions are summarized in Table 2. A Porapak Q 80/100 column was utilized, the flow rates of the carrier gas and hydrogen were 30 mL/min, respectively, and the air was set at 300 mL/min. The temperatures of sample inlet, oven, and detector were set at 70 °C, 120 °C, and 320 °C, respectively, and high purity nitrogen (99.999%) was used as carrier gas.

### 2.2. Quality Control

For the quality control, the N_2_O analysis was tested using gas standards. Thus, standard gases containing 1, 3, 5 and 10 ppm N_2_O (RIGAS, Daejeon, Korea) were transferred from the standard gas cylinders to a Tedlar bag (SKC, Washington, PA, USA) with a volume of 10 L. The Tedlar bag used in the transfer was previously purged three times with high purity nitrogen (99.999%) in order to minimize the effect of any remaining infinitesimal ingredients in the Tedlar bag during the analysis of the standard gases [19].

The four standard gases were analyzed more than five times, and then the linearity was confirmed by preparing calibration curves. The standard gas with the same concentration was repeatedly analyzed more than 10 times to evaluate the repeatability. Methods for obtaining the Detection Limit (DL) include those based on visual evaluation, signal to noise, and the slope of the standard deviation and calibration curve of the response. The method base on the slope of the standard deviation and calibration curve of the response was applied in this study. This method involves dividing the standard deviation of the response by the slope of the calibration and then multiplying by 3.3, as shown in Equation (1) [20]. The standard deviation was used, in which standard gas of 1 ppm was analyzed 5 times repeatedly and measured, and the slope of calibration curve was applied for confirming the detection limit:

(1) DL=3.3×∂/S

where ∂ is the standard deviation of the response and S is the slope of the calibration curve

### 2.3. Sampling and Analysis Method at Combustion Facilities

**Figure 1.**
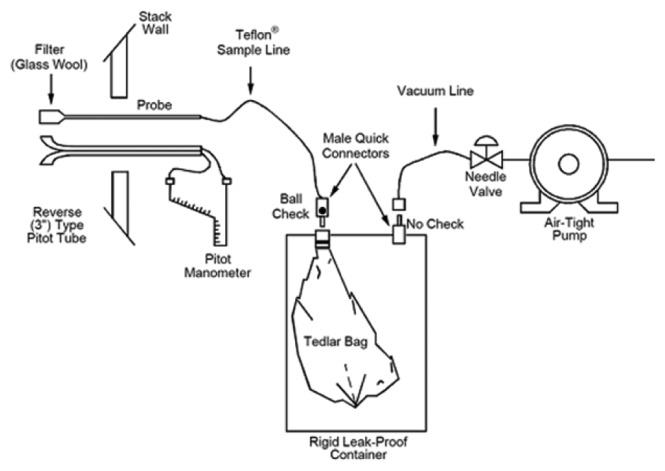
Diagram of the greenhouse gas sampling system.

EPA Method 18 [21] was applied for sampling the greenhouse gases that were emitted from fossil fuel combustion facilities. A schematic diagram is shown in Figure 1 below. Because the exhaust gases of a combustion facility are generally emitted with a velocity of more than 10 m/sec and at temperatures over 100 °C, the sampling pipe was manufactured with stainless steel that can endure such flow rates and temperatures [22]. A Vacuum Lung sampler (Acen, Suwon, Korea) that samples a specimen by utilizing a pressure difference created by applying a sound pressure in the inner part was used for the specimen sampler and a 10 L Tedlar bag (SKC) was used as the specimen collection container [23]. In the case of analysis of N_2_O in the exhaust gases from a combustion facility, moisture removal from the sample is the most important factor, making the moisture remover essential [7]. Therefore, a gas conditioner (JCC, JPES, Wiener Neustadt, Austria) and molecular sieve trap (Sigma-Aldrich) were connected to the front part to remove moisture. Four samples of exhaust gas in total were collected in an interval of 20 min; the collected samples were stored in black bags to minimize the losses caused by sunlight, and they were then analyzed three times using gas chromatography and photoacoustic spectroscopy in the laboratory.

### 2.4. Accuracy and Response Time of PAS

Taking into account of the fact that PAS can be used for continuous measurements, increased response times and decreased response times according to changes in the N_2_O standard gas concentrations (1, 5, 10 ppm) were analyzed separately. Increased response time was set at a standard time when the analyzer reached 95% of the target concentration when changing from a low concentration to a high concentration; decreased response time was set at a standard time when the analyzer reached 5% of the target concentration when changing from a low N_2_O concentration to a high N_2_O concentration [24]. Conformity among the measurements was judged by measuring the same sample repeatedly to confirm its accuracy. Accuracy is suggested by the percentage of standard deviation for the average of the measured value, or is expressed by the relative standard deviation [25].

## 3. Results

### 3.1. Drawing of the Calibration Curve

**Figure 2.**
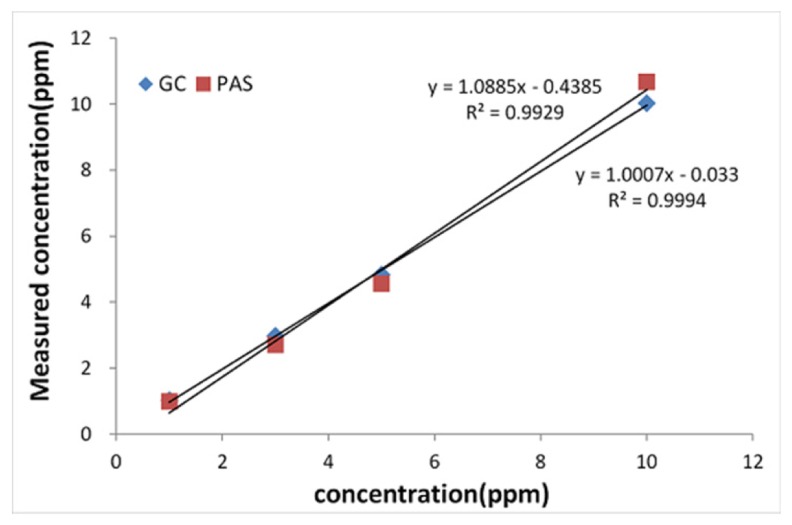
Calibration curve by GC and PAS.

Four standard gases with different concentrations (1, 3, 5, 10 ppm) were utilized for plotting the calibration curves for each N_2_O measurement method. The results are shown in Figure 2 below. The calibration curve that was obtained using PAS is expressed by a first degree equation, that is y = 1.0885x − 0.4385, R^2^ = 0.9929. In case of GC the calibration curve is expressed by the equation y = 1.0007x − 0.033, R^2^ = 0.9994. The linearity of PAS and GC are confirmed by their R^2^ = 0.99 values, and it is confirmed that the linearity of GC is superior to that of PAS.

### 3.2. Repeatability and Detection Limit

Standard gas (RIGAS) with a concentration of 10 ppm was repeatedly analyzed 10 times to confirm repeatability. The results are shown in Table 3. For PAS, the average value is evaluated for 10.46 ppm, the standard deviation for 0.12 ppm, and the relative standard deviation for 1.12%, respectively. For GC, the average value was evaluated at 10.00 ppm, the standard deviation at 0.02 ppm, and the relative standard deviation at 0.23%, respectively. The relative standard deviation of PAS is somewhat higher than that of GC, but the result of PAS is similar within 1%, which is the repeatability result from a prior study [2]. For both of the methods, the repeatability was measured at lower than 3.0%, which is suggested by ISO 1564 to indicate excellent repeatability.

The measurement of the detection limit was performed by applying the method based on the slope of the standard deviation of the response and calibration curve. The standard deviation of the measured values when 1 ppm was repeatedly analyzed five times was 0.008 for PAS and 0.023 for GC. The slope were calculated at 1.0885 in PAS and 1.0007 in GC, respectively, as shown in Figure 2, which is applied to calculating a detection limit; the detection limit was determined to be 0.025 ppm in PAS and 0.074 ppm in GC, respectively. A prior study [1] reported that the detection limit of PAS was 0.007 ppm and that of GC was 0.030 ppm, respectively, which are 3.6 and 2.5 times lower than compared to the detection limits in this study which was considered to be a very minute difference. Such differences in detection limits may result from the kinds of measuring devices and gases used,

### 3.3. Analysis and Comparison of the Exhaust Gases from Fossil Fuel Combustion

Four exhaust gases sampled from combustion facilities were analyzed three times, and the results are listed in Table 4. For PAS, the range of average concentrations went from a minimum of 32.40 ppm to a maximum of 33.43 ppm, and the average was 33.03 ppm. For GC, the range of average concentrations was from a minimum of 32.91 ppm to a maximum of 33.23 ppm, and the average was 33.07 ppm. The difference in average concentrations between PAS and GC is within 1%, showing very similar measurements. The standard deviation of the averaged concentrations was calculated at 0.34 ppm in PAS and 0.12 ppm in GC, and the relative standard deviation was calculated at 1.04% and 0.37%, respectively, which are excellent results. The relative standard deviation of PAS is a little higher than that of GC, which is very similar to the quality control result using standard gases.

### 3.4. Accuracies and Response Times per Concentration with PAS

**Figure 3.**
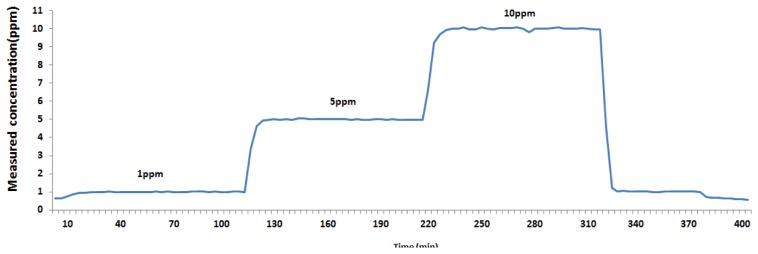
Trend of the concentration changes in PAS.

In order to identify the accuracy and response time characteristics per N_2_O concentration, a test was conducted by dividing N_2_O concentrations into 1, 5, and 10 ppm, and the results are summarized in Figure 3. The accuracy provides valuable information for judging the conformity among measured values by measuring the same sample repeatedly. The conformity may be expressed for a range of measured data or standard deviation, and it is often expressed as relative standard deviation in the case of repeated experiments [25]. The results for the evaluation of accuracy per concentration show that the standard deviation is 0.01 at 1 ppm concentration, 0.02 at 5 ppm, and 0.08 at 10 ppm, respectively, and the relative standard deviation is 1.25%, 0.45%, and 0.81% at 1, 5, and 10 ppm, respectively. This means the relative standard deviation of a low concentration of 1 ppm is higher than at higher concentrations of 5 and 10 ppm.

Since PAS is a method that can be used to measure continuously, it should respond sensitively to N_2_O concentration changes during on site measurements. The initial response time of PAS was 3 min and 26 s, but an increased response time pursuant to each of the concentration changes was measured by increasing the concentration of the standard gases in the order of 1 ppm, 5 ppm, and 10 ppm, respectively. These results show that a response time in which the injected concentration reached 95% of the target concentration and then stabilized, was confirmed to be 6 min and 52 s. By reducing the concentration from 10 ppm to 1 ppm and reaching 5% of the target concentration, the result is also shown to be 6 min and 52 s, confirming that the increased response time when changing from a low concentration to a high concentration, and the decreased response time when changing from a high concentration to a low concentration are both within 7 min and equal to each other. This is a slightly longer response time than those of the Fourier Transform Infrared Spectroscopy (FTIR) or amperometric methods, which have a response time of 50 s [1], but it is sufficient time for the continuous measurement criteria suggested by the atmospheric pollution process test, so it may be judged as usable for continuous measurements. Therefore, we believe that PAS is suitable for conducting continuous measurements at a large waste incineration facility where there is deviation in the concentrations of greenhouse exhaust gases, because it is easy to analyze trends of whole concentration changes.

### 3.5. Comparison between PAS and GC

The performance of PAS and GC were compared based on the laboratory and on site test results, which are summarized in Table 5. For measurement precision, PAS is inferior to GC, but for mobility PAS is superior. Also, PAS has a response time within 3 min and 30 s, which is quicker than the 5 min of GC. It has been reported in prior studies that PAS was able to evaluate site-measured data quicker in real time than GC [16].

## 4. Conclusions

In this study, linearity, accuracy, and response time were compared and evaluated by utilizing a PAS and GC method, N_2_O measurements, and actual exhaust gases emitted from fossil fuel combustion facilities that were collected and their characteristics analyzed.

First, in accuracy control by using standard gases, linearity was evaluated by plotting calibration curves. Repeatability was analyzed by repeatedly measuring a 10 ppm standard gas 10 times. The detection limits of PAS and GC were calculated by applying the method based on the standard deviation of responses and the slope of calibration curves.

R^2^ of PAS was evaluated at 0.9929 and that of GC at 0.9994, showing that both these methods have excellent linearity. The linearity analysis results show that the relative standard deviations of PAS and GC are 1.12% and 0.23% respectively, which are lower than the 3.0% reproducibility that is suggested from ISO 11564, thus showing excellent reproducibility. The detection limits of PAS and GC are determined to be 0.025 ppm and 0.074 ppm, respectively.

Second, exhaust gases collected using a Lung Sampler and Tedlar bag were analyzed. The results show that the average concentrations measured by PAS and GC are approximately 33.03 ppm and 33.07 ppm, respectively, which are similar to each other. Taking into account the merit that PAS makes it possible to conduct a continuous measurement, a changing trend by changing concentrations was analyzed as well. The results show that the standard deviations are 0.01 ppm, 0.02 ppm, and 0.08 ppm, respectively, and the relative standard deviations are 1.25%, 0.45%, and 0.81%, respectively. It was confirmed that the increased response time and the decreased response time pursuant to change of concentrations are both within 7 minutes in total.

The results of our analysis of the analytical characteristics of PAS and GC show that GC is superior in measurement accuracy, but PAS is better in cost efficiency, mobility, and convenience. PAS also makes it possible to conduct continuous measurements compared to GC, which has limitations in continuous measurement, so PAS is available to analyze entire concentration change trends.

The Mandatory Reporting Rule (MMR) in the USA regulates the execution of continuous sampling for 24 h a day at waste incineration facilities, since combustion characteristics vary according to the type and amount of waste being incinerated, and therefore the characteristics of the incinerated gas exhaust vary as well. Therefore, N_2_O measurements utilizing PAS, could be advantageous to check the exhaust characteristics of N_2_O per incineration facility and quantify the amount.

## Figures and Tables

**Table 1.** LSE N_2_O-4405 Analyzer Conditions.

**Analysis Condition**	**LSE N_2_O-4405 Analyser**
Sample flow rate	80 mL/min
Sample temperature	5∼25 °C
Pump speed	42%
Tqcl cycle scan	21∼25 °C
Current through QCL	0.65A
Temperature measurement cell	35 °C
Scan range	1277.847∼1279.548 cm^−1^

**Table 2.** Varian cp-3800 Conditions.

**Analysis Condition**		**Varian cp-3800**
Detector		ECD

Column		Porapack Q 80/100

Carrier gas		N_2_ (99.999%)

Flow	N_2_	20 mL/min
	H_2_	30 mL/min
	Air	300 mL/min

Temperature	Oven	70 °C
	Injector	120 °C

**Table 3.** Repeatability and detection limits of N_2_O analysis methods (units: ppm)

**Repeatability**		

**Number of Analysis**	**Photoacoustic Spectroscopy(PAS)**	**Gas Chromatography(GC)**
1	10.25	10.03
2	10.33	10.01
3	10.37	10.01
4	10.52	9.98
5	10.53	9.99
6	10.43	10.01
7	10.53	10.04
8	10.58	10.00
9	10.62	10.00
10	10.47	9.96

Mean	10.46	10.00
SD	0.12	0.02
RSD(%)	1.12	0.23

**Detection Limit**								

**Analysis Method**	**Measured Concentration**					**Mean**	**SD**	**DL**

	**1**	**2**	**3**	**4**	**5**			
PAS	0.989	0.977	0.986	0.996	0.997	0.989	0.008	0.025
GC	1.025	1.034	1.070	1.041	1.009	1.036	0.023	0.074

**Table 4.** Analysis of the exhaust gas concentrations with PAS and GC.

**Analysis Method**	**Sample Name**	**Measured Concentration (ppm)**				**SD**	**RSD (%)**

		**1**	**2**	**3**	**Mean**		
PAS	Sample 1	32.15	32.79	32.27	32.40	0.34	1.05
	Sample 2	32.93	33.60	32.91	33.15	0.39	1.18
	Sample 3	33.77	33.59	32.94	33.43	0.44	1.31
	Sample 4	33.33	32.92	33.11	33.12	0.21	0.62

Mean					33.03	0.31	1.04

GC	Sample 1	32.70	33.06	33.08	32.95	0.21	0.65
	Sample 2	32.97	32.86	32.89	32.91	0.06	0.17
	Sample 3	33.01	33.21	33.33	33.18	0.16	0.49
	Sample 4	33.20	33.29	33.20	33.23	0.05	0.16

Mean					33.07	0.12	0.37

**Table 5.** Analysis of the exhaust gas concentrations with PAS and GC.

**Division**	**PAS**	**GC**
Accuracy	Adequate	Good
Response time	Short(<3 min 30 s)	Long(>5 min)
Limit of detection	0.025 ppm	0.074 ppm
Mobility	Good	Poor
Ease of use	Good	Poor
Capital cost	High cost	Low cost
